# Identification of microRNAs as diagnostic biomarkers for atrial fibrillation: a systematic review and meta-analysis

**DOI:** 10.3389/fcvm.2023.1128708

**Published:** 2023-04-28

**Authors:** Ardian Rizal, Yoga Waranugraha, Adhika Prastya Wikananda, Yoga Yuniadi

**Affiliations:** ^1^ Department of Cardiology and Vascular Medicine, Saiful Anwar General Hospital - Faculty of Medicine Universitas Brawijaya, Malang, Indonesia; ^2^ Center for Cardiovascular Studies, Universitas Brawijaya, Malang, Indonesia; ^3^ Department of Cardiology and Vascular Medicine, Faculty of Medicine, Universitas Indonesia, National Cardiovascular Center Harapan Kita (Indonesia), Jakarta, Indonesia; ^4^ Department Cardiology and Vascular Medicine, Faculty of Medicine, Universitas Indonesia, Jakarta, Indonesia

**Keywords:** atrial fibrillation, genetic, micro-RNA (miRNA), biomarker, diagnostic

## Abstract

**Background:**

Genetic factors contribute to the AF pathophysiology by altering the structural and functional properties of proteins involved in different cellular activities. MicroRNAs (miRNAs), which take part in structural and electrical remodeling during the AF evolution, are important genetic elements that must be considered. The aim of study is to determine correlation between the expression of miRNAs and the development of AF, as well as to explain any potential importance of genetic factors in the AF diagnosis.

**Methods and Results:**

Online scientific databases, including Cochrane, ProQuest, PubMed, and Web of Science were used to conduct the literature search. The keywords were associated with or characterized the relationship between miRNAs and AF. The pooled sensitivity and specificity statistical parameters were analyzed using a random-effects model. The miRNAs had a combined sensitivity and specificity of 0.80 (95% CI = 0.70–0.87) and 0.75 (95% CI = 0.64–0.83) for the diagnosis of AF, respectively. The area under the SROC was 0.84 (95% CI = 0.81–0.87). The DOR was 11.80 (95% CI = 6.79–20.50). This study also revealed that miRNAs had a pooled PLR of 3.16 (95% CI = 2.24–4.45) and NLR of 0.27 (95% CI = 0.18–0.39) for the diagnosis of AF. The miR-425-5p demonstrated the highest sensitivity (0.96, 95% CI, 0.89–0.99).

**Conclusion:**

The meta-analysis revealed substantial connection between miRNA expression dysregulation and AF, supporting the potential diagnostic role of miRNAs. The miR-425-5p has potential role as a biomarker for AF.

## Introduction

The most frequent supraventricular arrhythmia, atrial fibrillation (AF), is associated with high rates of morbidity and death and significantly raises the risk of stroke, systemic thromboembolism, and heart failure (1, 2). The estimated prevalence of AF in adults is currently between 2% and 4%. Due to increased lifespan among the general population and a more intense screening for undiagnosed AF, a 2.3-fold increase is anticipated (2). Several well-known risk factors, such as male sex, older age, hypertension, diabetes mellitus, obstructive sleep apnea, heart failure, valvular heart disease, left atrial enlargement, and chronic obstructive pulmonary disease, play an essential role in the development of AF (3). In addition to those risk factors, family history is also thought to play a crucial role in the development of AF. Family history is associated with genetic factors, such as autosomal dominant inheritance, where epigenetic mechanisms can contribute to the AF pathophysiology by altering the structural and functional properties of proteins involved in different cellular activities (4, 5). MicroRNAs (miRNAs), which take part in structural and electrical remodeling during the AF evolution, are important genetic elements that must be considered (6).

Small non-coding RNAs known as miRNAs are encoded by nuclear DNA and transcribed by RNA polymerase II. Their main role is to control post-transcriptional gene expression by binding to complementary target sequences in messenger RNA (mRNA), which prevents the transcribed target from being translated or degraded (6). In other words, miRNAs play a role in the post-transcriptional regulation of protein expression and take part in the pathogenesis of the disease (7). The dysregulated miRNAs may be used as a new disease-specific diagnostic biomarker and treatment target (8, 9). The performance of miRNAs in detecting several cardiovascular diseases, including acute myocardial infarction, heart failure, and stroke, has been well-recognized (10–13). Several studies have investigated the connection between miRNAs and AF or AF recurrence following catheter ablation procedures in recent years (14–16). In total, 51 consistently dysregulated miRNAs linked to AF were identified (17). Beside the role of miRNA as diagnostic tolls of AF, several studies also conclude that miRNA can be used as Major Adverse Cardiovascular Events (MACE) in patient with AF (18). However, the performance of miRNA as the potential biomarker for AF diagnosis has not been widely explored. The goal was to determine whether there is a correlation between the expression of miRNAs and the development of AF, as well as to explain any potential importance of genetic factors in the AF diagnosis.

## Methods

### Study design

The Preferred Reporting Items for Systematic Reviews and Meta-Analyses (PRISMA) 2020 Statement was followed in this systematic review and meta-analysis study (19). Because this study was a systematic review and meta-analysis, patient-informed consent and ethical approval were not required.

### Literature search strategy

A conscientious literature search was conducted to find eligible original studies for this systematic review and meta-analysis. Online scientific databases, including Cochrane, ProQuest, PubMed, and Web of Science were used to conduct the literature search. The keywords were associated with or characterized the relationship between miRNAs and AF. The keywords were described or associated with the relationship between miRNAs and AF. We searched the potential articles with the following keywords: “micro-RNA” OR “micro RNA” OR “miRNA” AND “atrial fibrillation” OR “AF” OR “Afib” AND “diagnostic” OR “diagnosis” AND “sensitivity” OR “specificity.” The literature search was completed in November 2022.

### Eligibility criteria and quality of study assessment

The inclusion criteria of this systematic review and meta-analysis study were; (1) observational study; (2) exploring the diagnostic performance of miRNAs concentration for AF; (3) providing the sensitivity and specificity data of miRNAs in detecting AF. Articles were excluded according to the following criteria: (1) full-text unavailability; (2) not written in English; (3) non-human studies; (4) editorials; (5) letters to the editor, (6) article reviews; and (7) studies with inadequate information regarding diagnostic performance, sensitivity or specificity. QUADAS-2 tools for diagnostic studies were used to evaluate the quality of the included studies. It consists of four domains as follows: (1) patient selection, (2) the index test, (3) the reference standard, and (4) flow and timing (20).

### Data extraction

The data extraction included the following information: (1) first author's name, (2) publication year, (3) design, (4) enrolment period; (5) number of participants in the AF and sinus rhythm groups: (6) specimen; (7) miRNAs studied; (8) miRNAs quantification method; (9) miRNAs expression in AF; and (10) AF detection method. The number of participants with true-positive (TP), true-negative (TN), false-positive (FP), and false-negative (FN) results was also extracted. The authors calculated these values for investigations in which such information was not directly provided according to the associated sensitivity and specificity.

### Statistical analysis

For the statistical analysis, STATA 17.0 was utilized. Statistically significant was defined as *p*-value <0.05. The pooled sensitivity and specificity statistical parameters were analyzed using a random-effects model. The summary receiver operating characteristic (SROC) curve was used to assess the overall diagnostic efficacy (21). The hierarchical summary receive operating characteristic (HSROC) curve was used to validate bivariate model data The diagnostic odds ratio (DOR), positive likelihood ratio (PLR), and negative likelihood ratio (NLR) were also assessed (10). Using the heterogeneity index (*I*^2^) and the Cochran-Q test, the heterogeneity was evaluated (22). The significant heterogeneity was identified when the Cochran-Q test's *p*-value was less than 0.01 and the *I*^2^ value was greater than 50%. The potential publication bias of diagnostic research was evaluated using Deeks' funnel plot (23).

## Results

### Study selection

**Figure 1:**
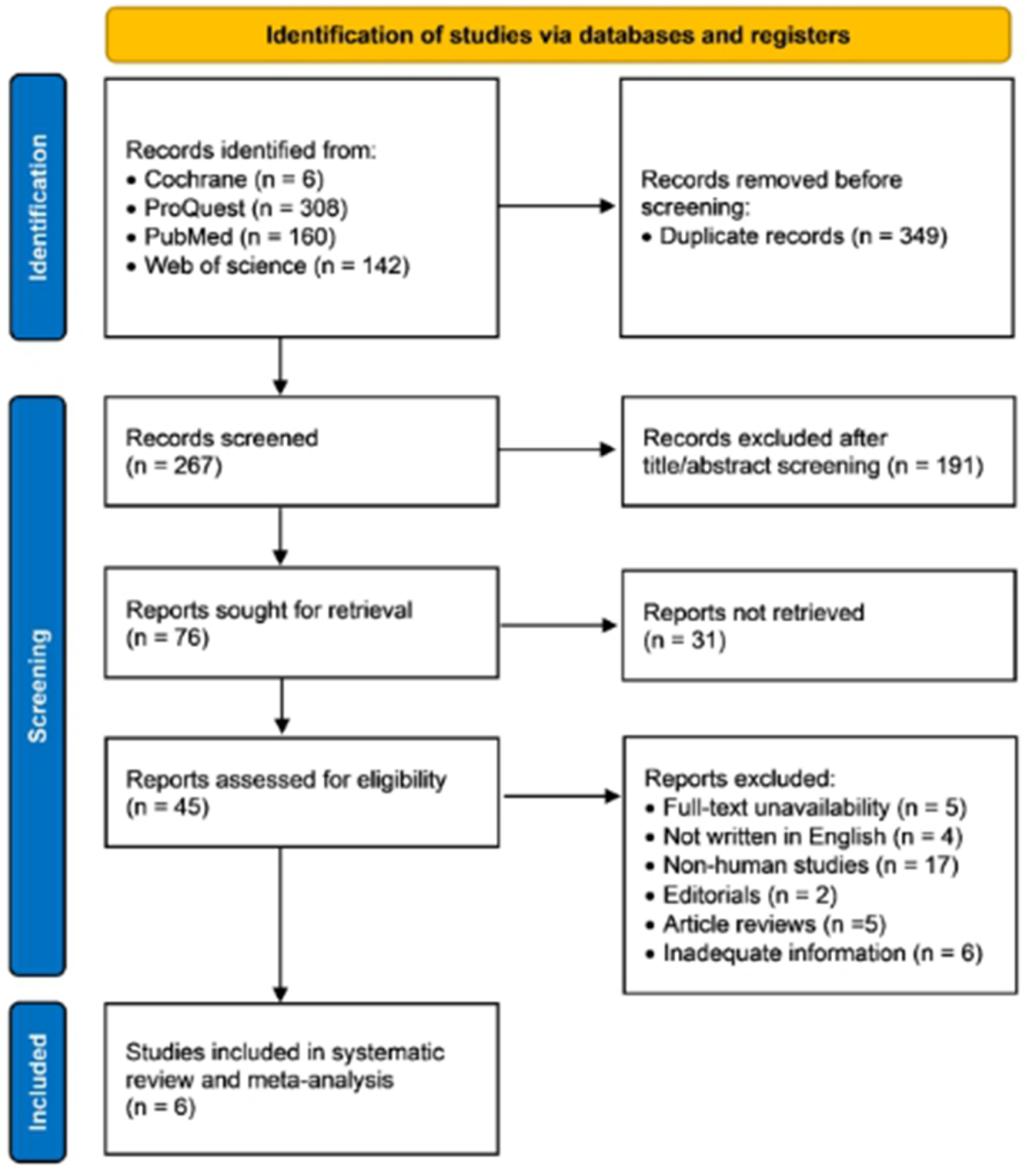
Flow diagram of the study selection process.

A total of 616 articles were identified from Cochrane (*n* = 6), ProQuest (*n* = 308), PubMed (*n* = 160), and Web of Science (*n* = 142) databases, among which 349 records were excluded due to duplications. During the title/abstract screening, we excluded 191 records due to mismatch with our purposes. In the next step, 31 reports were not retrieved. The remaining 45 articles were included in the further selection, and 39 articles were excluded due to: (1) full-text unavailability (*n* = 5); (2) not written in English (*n* = 4); (3) non-human studies (*n* = 17); (4) editorials (*n* = 2); article reviews (*n* = 5), and inadequate information (*n* = 6). Finally, 6 studies evaluating the diagnostic performance of miRNAs expression in AF were included in the meta-analysis. Figure 1 provides a schematic representation of the literature review and the selection criteria for the study.

### Baseline characteristics

**Figure 2:**
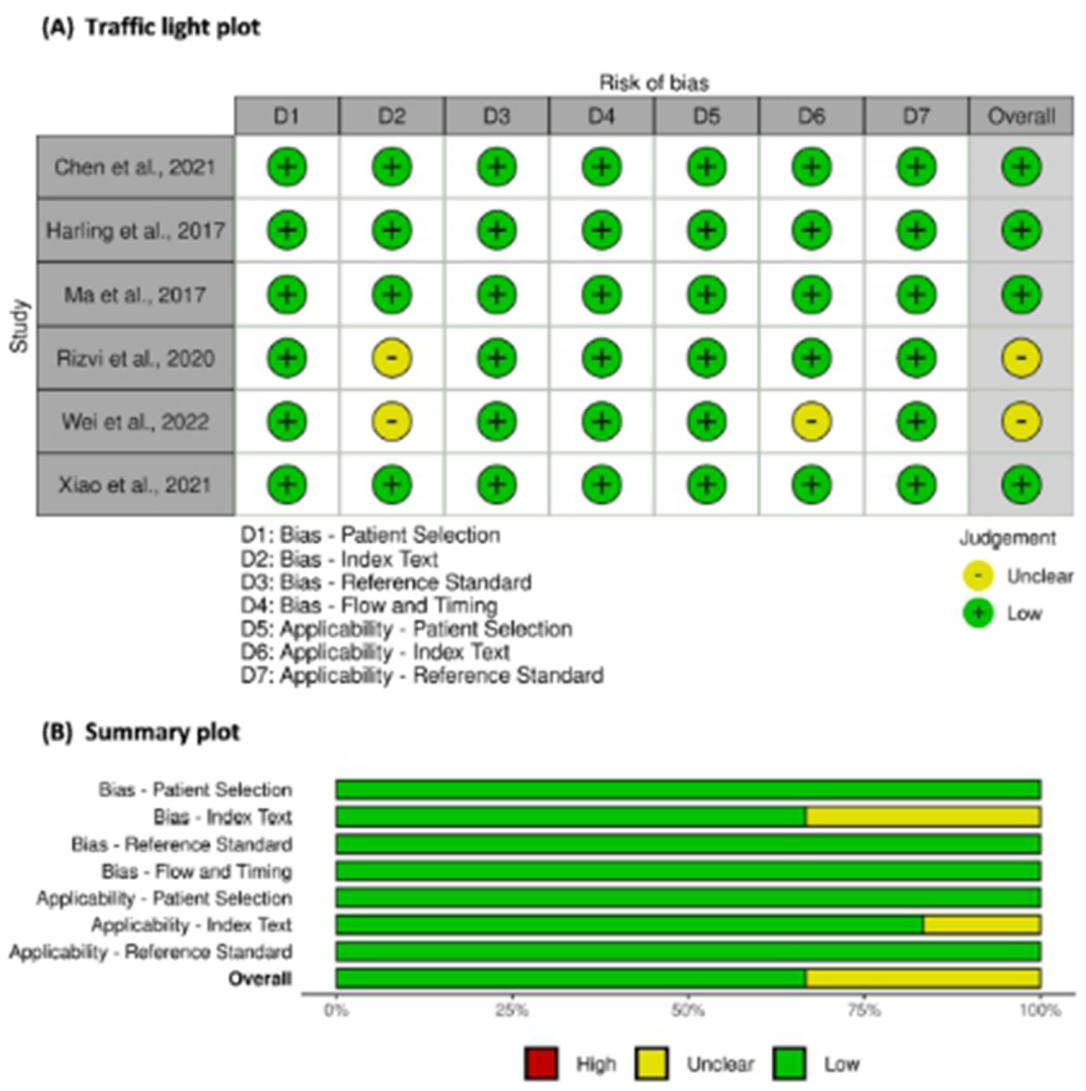
Risk of bias assessment of studies using QUADAS-2. (**A**) Traffic light plot and (**B**) Summary plot.

A total of 1,065 participants, including 547 and 518 participants in the AF and SR groups from 6 studies, were included in the data analysis. Four studies used plasma samples, whereas two studies used serum for miRNA quantification. MiRNAs were measured using quantitative reverse transcription polymerase chain reaction (qRT-PCR). The miRNA studied were miR-21, miR-483-5p, miR-208, miR-499, miR-29, miR-425-5p, and hsa-miR-4443. The baseline characteristics of the included studies are summarized in Table 1. According to the QUADAS-2 assessment, studies with a high risk of bias were not found (Figure 2).

**Table 1:** Baseline characteristics of the included studies.

Study	Country	Enrolment period	AF (n)	SR (n)	Specimen	miRNA studied	miRNA quantification method	miRNA expression in AF	AF detection method
Chen et al., 2021	China	2016 to 2017	60	60	Plasma	miR-21	qRT-PCR	Overexpressed	ECG
Harling et al., 2017	UK	November 2010 to September 2011	13	21	Serum	miR-483-5p	qRT-PCR	Overexpressed	Holter monitoring
Ma et al., 2017	China	November 2010 to September 2014	64	76	Plasma	miR-208	qRT-PCR	Overexpressed	ECG
						miR-21	qRT-PCR	Overexpressed	
						miR-499	qRT-PCR	Overexpressed	
Rizvi et al., 2020	USA	April 2013 to November 2014	34	56	Plasma	miR-29	qRT-PCR	Underexpressed	ECG
									Telemetry-based ECG monitoring
Wei et al., 2022	China	October 2018 to October 2019	89	89	Plasma	miR-425-5p	qRT-PCR	Underexpressed	ECG
Xiao et al., 2021	China	January 2017 and May 2020	123	100	Serum	miR-4443	qRT-PCR	Underexpressed	ECG
									Holter monitoring

AF, atrial fibrillation; ECG, electrocardiogram; miRNA, micro-RNA; SR, sinus rhythm; qRT-PCR, quantitative reverse transcription polymerase chain reaction.

### Diagnostic performance of MiRNAs for AF

**Figure 3:**
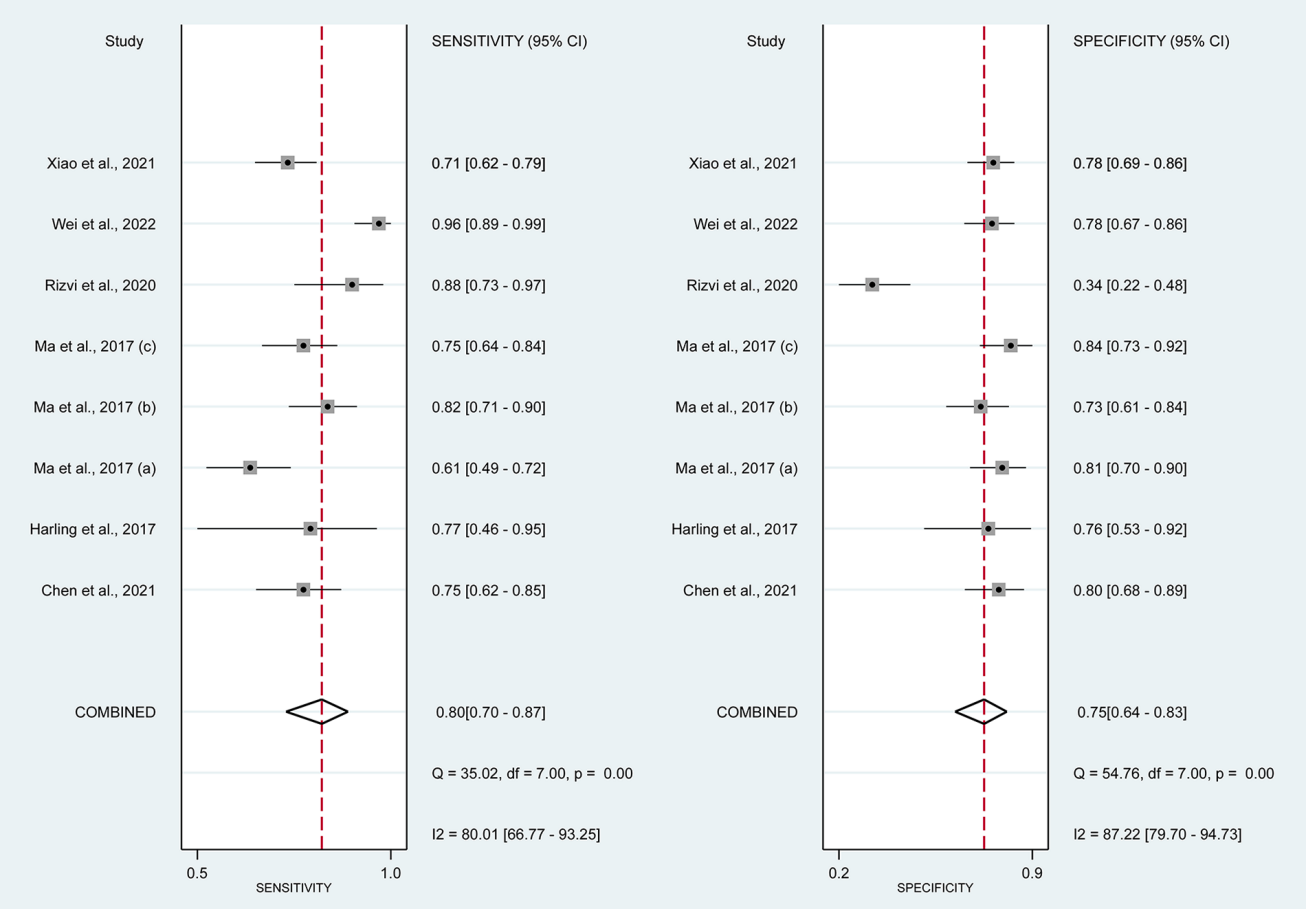
Forrest plot of estimates of sensitivity and specificity. MiRNAs had combined sensitivity 0.80 (95% CI = 0.70–0.87, *p* < 0.01) and specificity 0.75 (95% CI, 0.64–0.83; *p* < 0.01).

**Figure 4:**
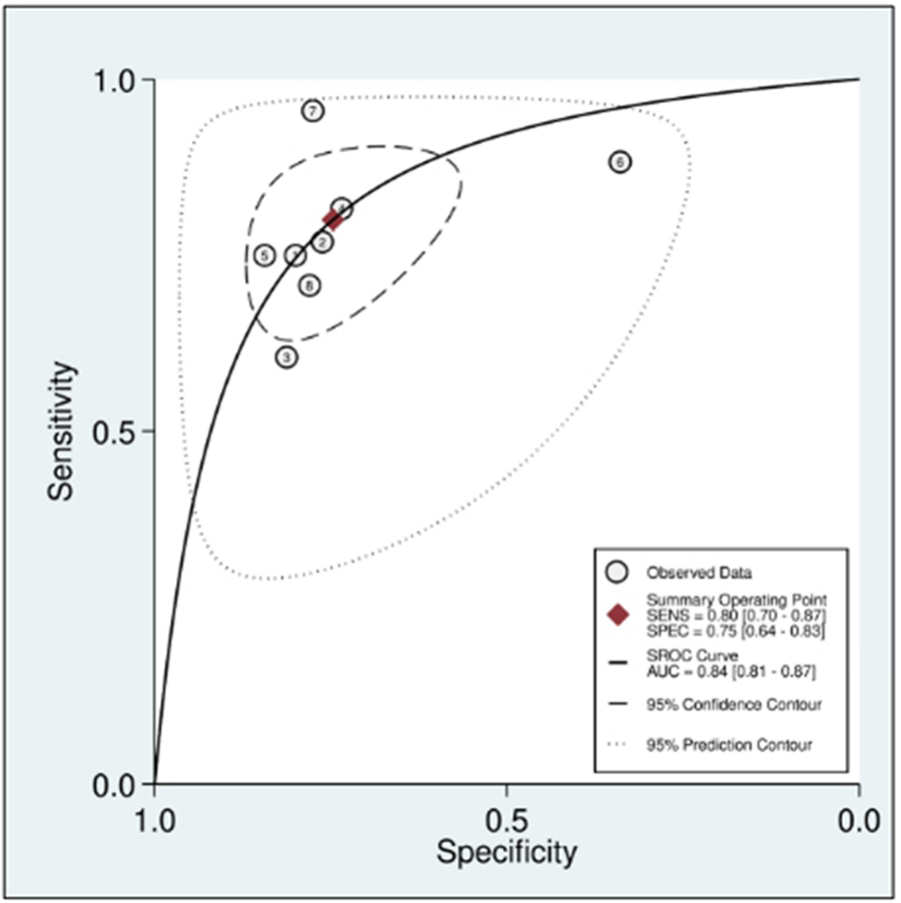
Summary receiver operating characteristic (SROC) curve.

**Figure 5:**
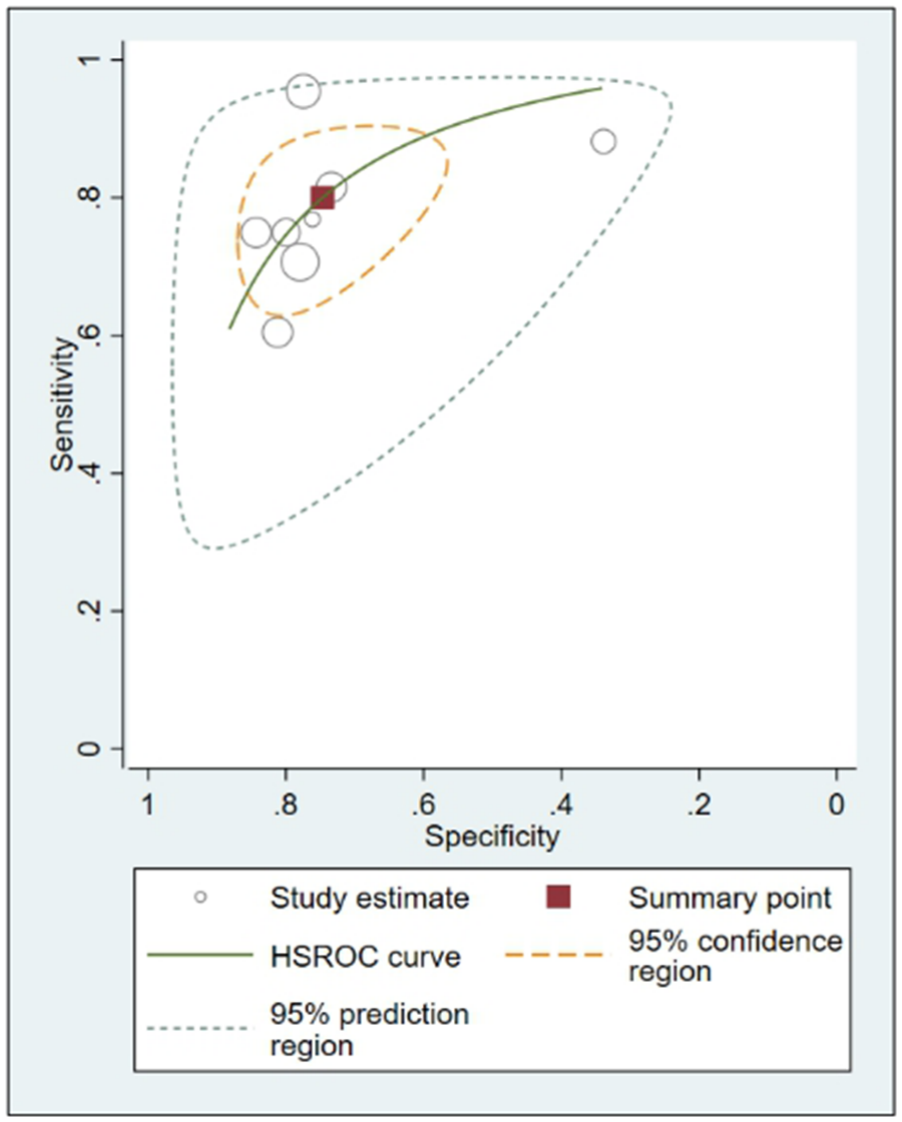
Hierarchical summary receiver-operating characteristic (HSROC) curve.

**Figure 6:**
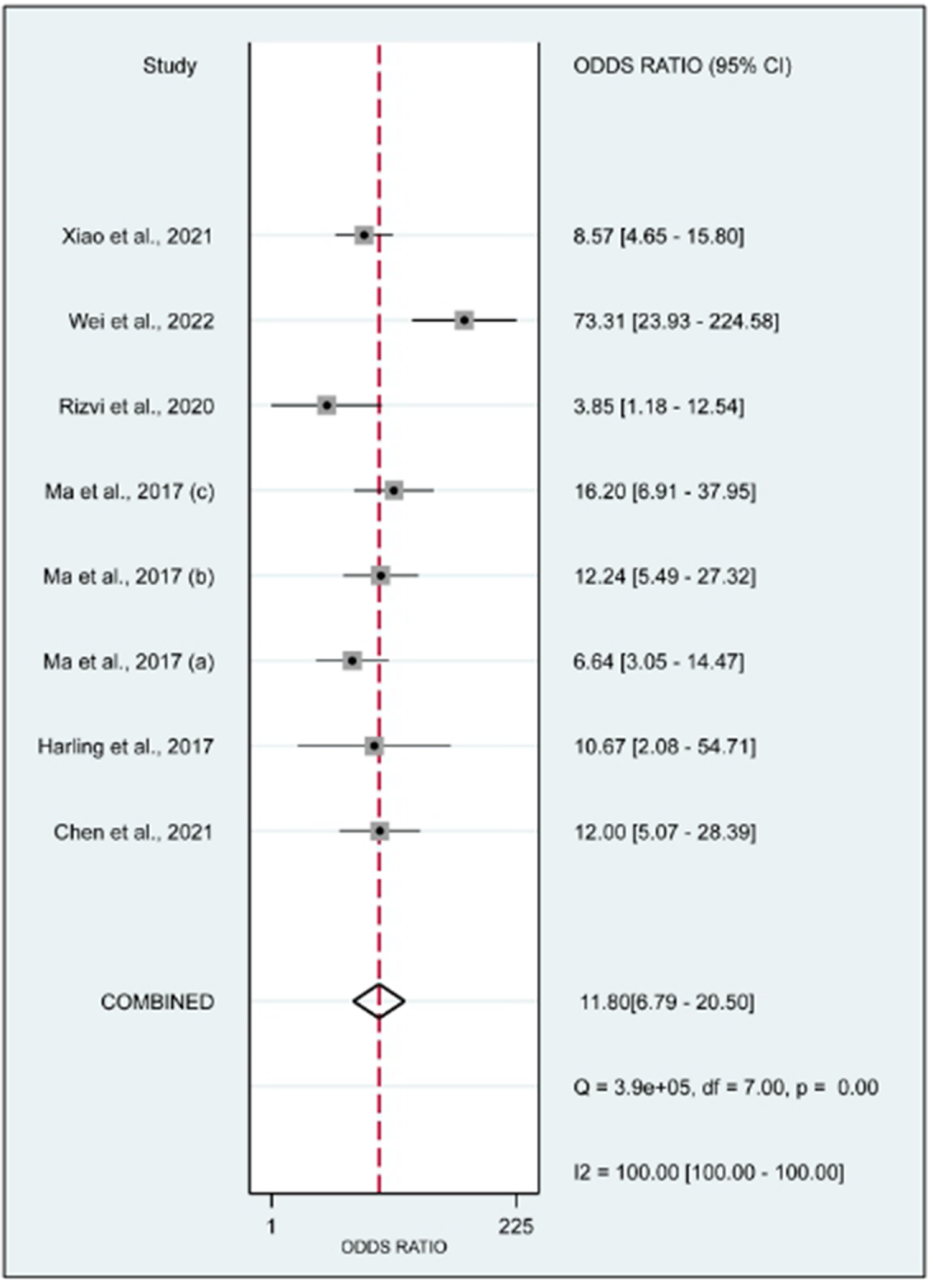
Forrest plot of the diagnostic odds ratio. The DOR was 11.80 (95% CI, 6.79–20.50; *p* < 0.01).

**Figure 7:**
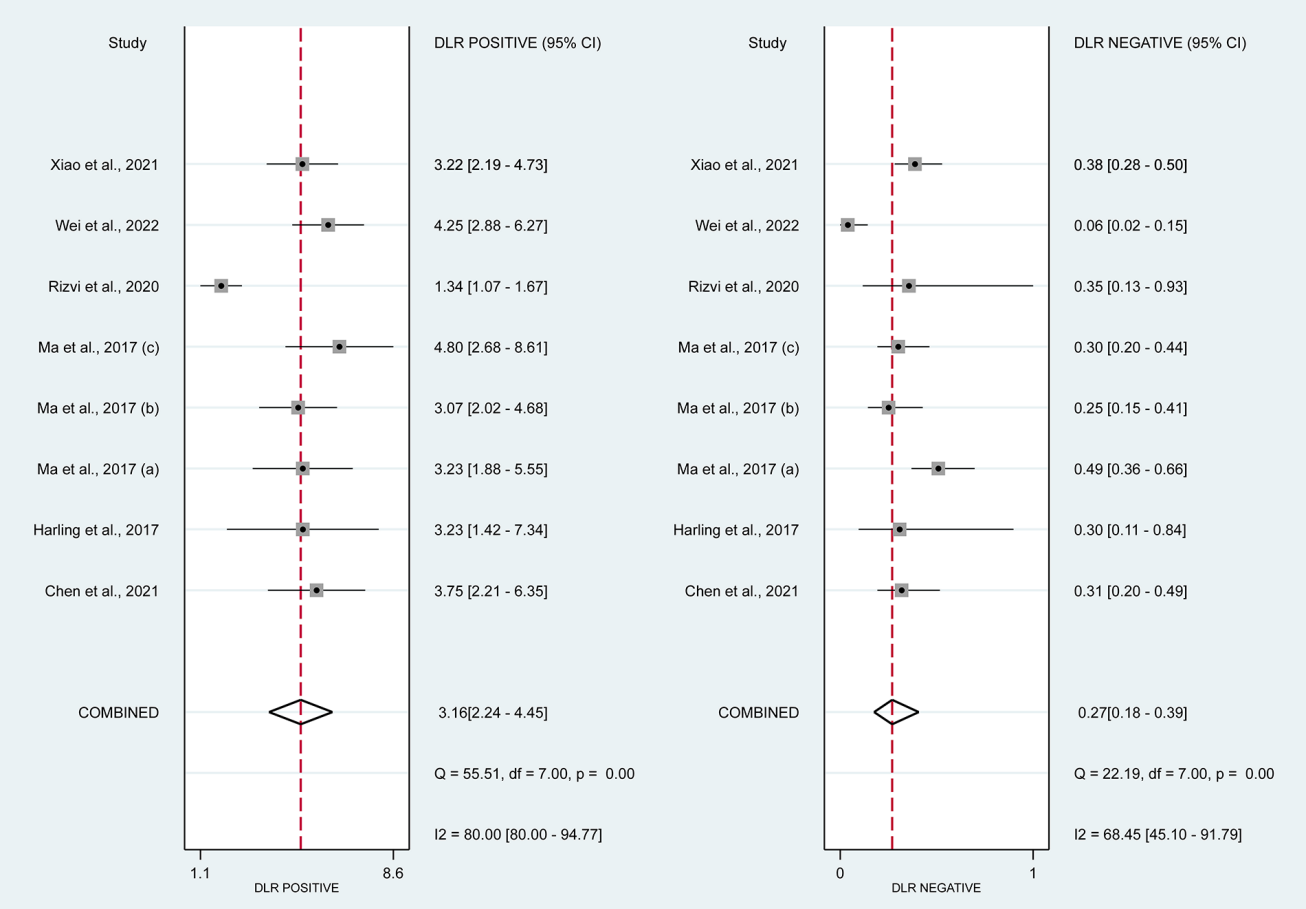
Forrest plot of estimates of positive likelihood ratio and negative likelihood ratio. The PLR was 3.16 (95% CI, 2.24–4.45; *p* < 0.01) and The DLR was 0.27 (95% CI, 0.18–0.39; *p* < 0.01).

**Figure 8:**
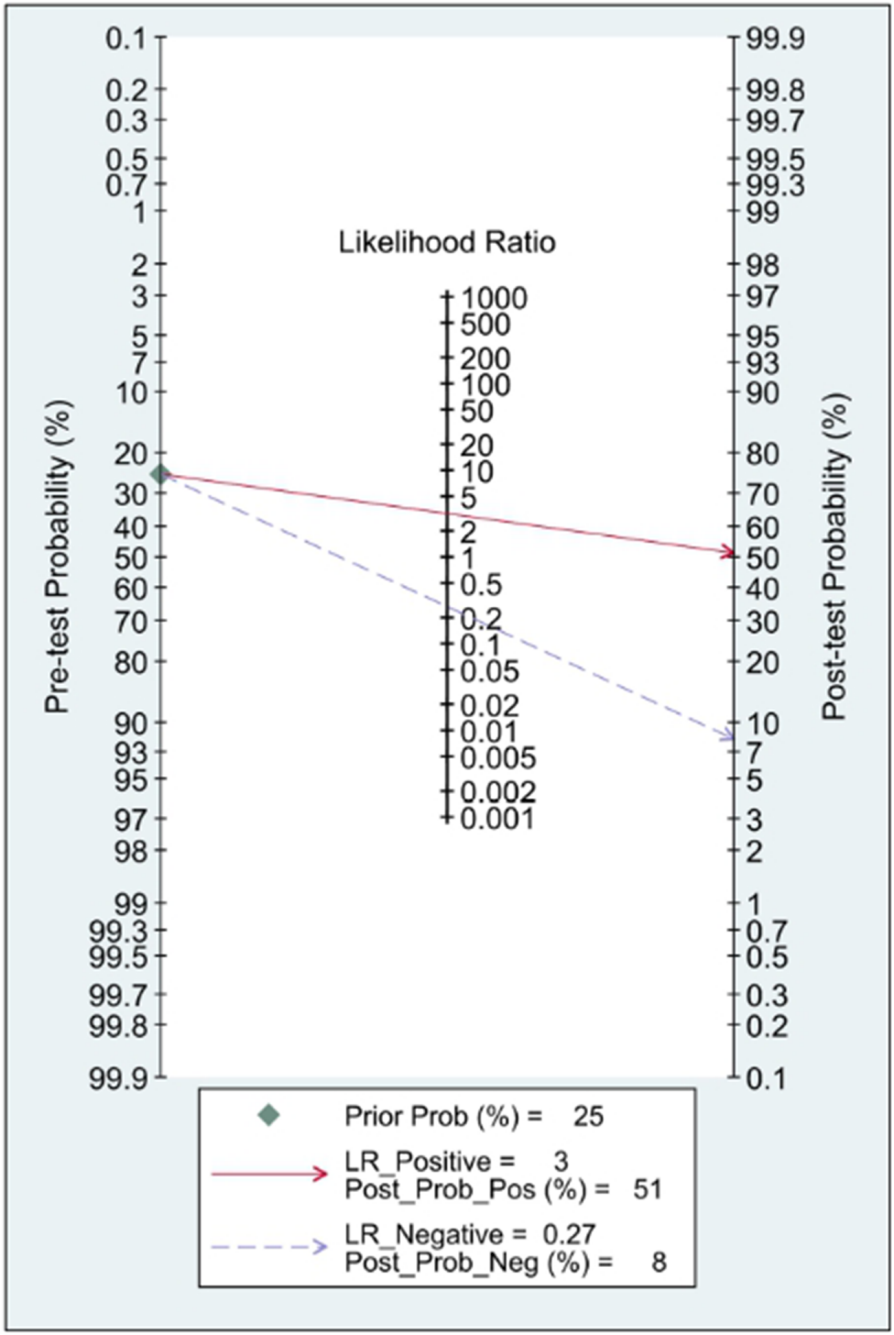
Fagan's Nomogram for assessment of post-test probabilities.

In this systematic review and meta-analysis study we explored the diagnostic performance or miRNAs for AF that included several parameters such as sensitivity, specificity, area under curve of SROC, PLR, and NLR. MiRNAs had a combined sensitivity and specificity of 0.80 (95% CI = 0.70–0.87) and 0.75 (95% CI = 0.64–0.83) for the diagnosis of AF, respectively (Figure 3). Using SROC curve analysis, the combined diagnostic performance of miRNAs was evaluated (Figure 4). The area under the SROC was 0.84 (95% CI = 0.81–0.87). In the results given by the HSROC model (Figure 5 and Supplementary Figure S1), the *β* (beta) estimation and the 95% CI were −0.02 (95% CI = −0.82–0.79), and z = −0.04, *p* = 0.97 (*p* > 0.5). The *λ* (lambda) estimation and the 95% CI were 2.47 (95% CI, 1.91–3.02). Those results are consistent. Additionally, the DOR was assessed (Figure 6). The DOR was 11.80 (95% CI = 6.79–20.50). This study also revealed that miRNAs had a pooled PLR of 3.16 (95% CI = 2.24–4.45) and NLR of 0.27 (95% CI = 0.18–0.39) for the diagnosis of AF (Figure 7). Figure 8 demonstrates the likelihood of correctly diagnosing a patient with AF is 51% if the miRNA test yields a positive result. The likelihood of misdiagnosing a patient without AF as having AF is 8% if the miRNA test results are negative.

### Publication bias

**Figure 9:**
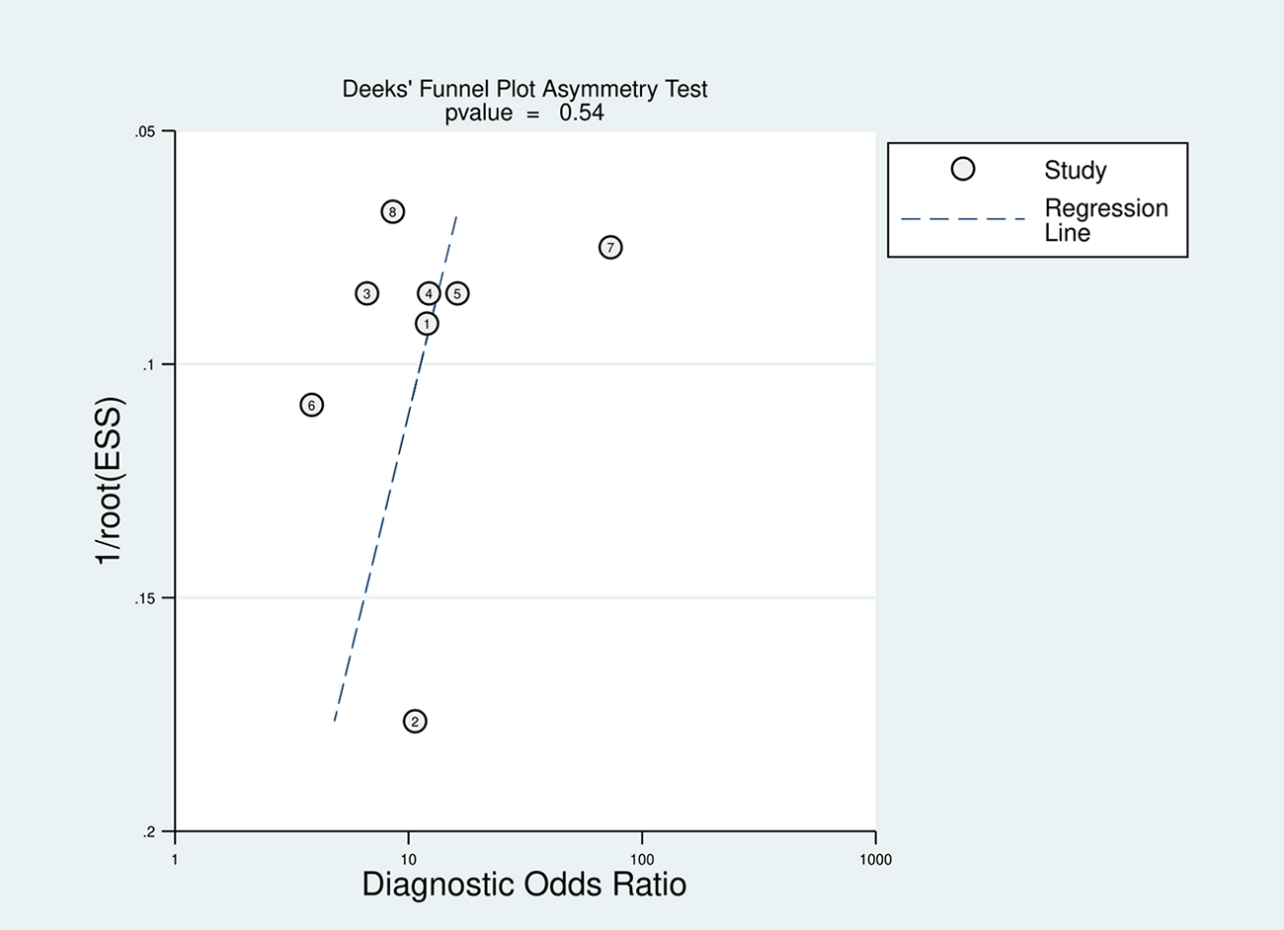
Deeks’ funnel plot asymmetry test was used to assess the publication bias.

Using STATA 17.0, Deeks' funnel plot was created to assess the underlying publishing bias. That plot revealed a *p*-value of 0.54, indicating a low publication bias probability (Figure 9).

## Discussion

MiRNAs regulate essential target genes and perform crucial roles in the development of AF. Multiple experimental studies have demonstrated that miRNAs have a role in atrial electric remodeling in AF by targeting essential genes. Multiple strategic miRNA-related pathways, including Ca2+-dependent signaling pathways, inflammatory and immunological pathways, apoptotic and cycle pathways, have been identified, indicating that miRNAs are likely to be the therapeutic target for AF (24). Multiple studies have examined the potential use of miRNAs as biomarkers for AF. In this study, the relationship between dysregulation of miRNAs and the occurrence of AF was originally established using a pooled analysis of previously published data. Quantitative approaches were then utilized to corroborate this association. In addition, we compared the diagnostic value of miRNAs in various studies and discovered that miR-425-5p reported by Wei et al. (2022) (25) demonstrated the highest sensitivity (0.96, 95% CI, 0.89–0.99, Figure 3) and diagnostic value (73.31, 95% CI, 23.93–224.58, Figure 5), suggesting the potential role of miR-425–5p as a biomarker for AF. This meta-analysis revealed a substantial connection between miRNA expression dysregulation and AF, supporting the potential diagnostic role of miRNAs.

AF known to be initiated and perpetuated by electrical and structural remodeling of the atrium. In this meta-analysis, 2 studies found that over expression of miR-21 had diagnostic role of AF. Previous study found that miR-21 was found to be higher in atrial tissue patient with AF compared to those in sinus rhythm. Its over expression decreasing L-Type Ca2+ current expression, which is the process of electrical remodeling (26). Another study found that higher miR-21 concentration had correlation with atrial tissue fibrosis, the key process of atrial tissue structural remodeling (27). Harling et al. found used miR-483-5p in their study. This type of miRNA had a role of manipulating cytokine signaling by targeting SOCS3 (suppressor of cytokine signaling-3). SOCS3 previously found to be important in the development of metabolic syndrome by increasing the risk of obesity and insulin resistance (28).

Other mechanisms of how different miRNA act on initiation and perpetuation are summarized in Table 2.

**Table 2:** Type of MiRNAs and its possible mechanism of Developing AF.

Study	miRNA studied	miRNA expression in AF	Mechanism
Chen et al., 2021	miR-21	Overexpressed	Increasing cardiac fibrosis (26)
Harling et al., 2017	miR-483-5p	Overexpressed	Obesity and insulin resistance (risk factor of AF) (27)
Ma et al., 2017	miR-208	Overexpressed	Myocytes hypertrophy, decreasing myocyte contraction and cardiac conduction abnormalities
	miR-21	Overexpressed	
	miR-499	Overexpressed	
Rizvi et al., 2020	miR-29	Underexpressed	Increasing cardiac fibrosis (29)
Wei et al., 2022	miR-425-5p	Underexpressed	Increasing cardiac fibrosis (30)
Xiao et al., 2021	hsa-miR-4443	Underexpressed	Increasing myocardial fibroblast proliferation (31)

Based on the first systematic review of its kind to investigate the miRNA expression markers of AF (17), the previous study need for a meta-analysis to produce specific and useful miRNA data stems from the wide variety of technology platforms and inconsistencies in sample sources. A total of 51 miRNAs showed systematic abnormal expression across all studies. A total of five microRNAs (miRNA-328, miRNA-223-3p, miRNA-21, miRNA-29b, and miRNA-1-5p) were found through additional investigation as possible biomarkers of atrial fibrillation. MiRNA-21 was identified as being overexpressed in two studies, both of which were included in the meta-analysis (32–34). We recommend further study of miRNA-21 to identify more specific miRNA that can be utilized as a more accurate diagnostic tool for atrial fibrillation.

A single biomarker, miRNA-328-3p, was found to be strongly related with an elevated risk of AF in another meta-analysis investigation. This finding suggests that miR-328-3p was crucial in the diagnosing process and could be a significant and effective biomarker for detecting AF (35). However, miR-328-3p has also been linked to a wide variety of other diseases and disorders. Numerous studies have shown that miR-328-3p can inhibit tumor growth (36). Several limitations were discovered in our meta-analysis. Some miRNAs may be differentially expressed between paroxysmal and chronic AF. However, this was not investigated in the current study. Moreover, the examined group showed heterogeneities in comorbidities such valve disease and rheumatic heart disease, which may have served as confounding factors, adding to the heterogeneity of miRNA expression.

Recently, a meta-analysis published in early 2023 revealed strong correlation between circulating miRNA and Atrial fibrillation. Unlike our study, this study did not evaluate diagnostic performance including pooled sensitivity and specificity. They analyzed each type of the OR of each miRNA and then measured the overall OR. The overall OR was 2.51 (95% CI, 1.99-3.16). The miRNA—150 had highest OR (3.77; 95% CI, 1.50–9.46) (37).

## Data Availability

The original contributions presented in the study are included in the article, further inquiries can be directed to the corresponding author.
