# Supplementary material for: Identification of microRNAs as diagnostic biomarkers for atrial fibrillation: a systematic review and meta-analysis
Source: Front Cardiovasc Med. 2023 Apr 28;10:1128708. doi: 10.3389/fcvm.2023.1128708 (PMC10181881; doi:10.3389/fcvm.2023.1128708)
Supplement: Supplementary file 1 [file Table1.docx]

Supplementary table 1. QUADAS.

| QUADAS | Chen et al., 2021 | Harling et al., 2017 | Ma et al., 2017 | Rizvi et al., 2020 | Wei et al., 2022 | Xiao et al., 2021 |
| --- | --- | --- | --- | --- | --- | --- |
| Was the spectrum of patient representative of the patients who will receive the test in practice? | 1 | 1 | 1 | 1 | 1 | 1 |
| Were selection criteria clearly described? | 1 | 1 | 1 | 1 | 1 | 1 |
| Is the reference standard and index test short enough to be reasonably sure that the target condition did not change between the two tests? | 1 | 1 | 1 | 1 | 1 | 1 |
| Is the time period between reference standard and index test short enough to be reasonably sure that the target condition did not change between the two tests? | 1 | 1 | 1 | 1 | 1 | 1 |
| Did the whole sample or a random selection of the sample, receive verification using a reference standard of diagnosis? | 1 | 1 | 1 | 1 | 1 | 1 |
| Did patients receive the same reference standard regardless of the index test result? | 1 | 1 | 1 | 1 | 1 | 1 |
| Was the reference standard independent of the index test (i.e. the index test did not form part of the reference standard)? | 1 | 1 | 1 | 1 | 1 | 1 |
| Was the execution of the index test described in sufficient detail to permit replication of the test? | 1 | 1 | 1 | 0 | 0 | 1 |
| Was the execution of the reference standard described in sufficient detail to permit its replication? | 1 | 1 | 1 | 1 | 1 | 1 |
| Were the index test results interpreted without knowledge of the results of the reference standard? | 1 | 1 | 1 | 1 | 1 | 1 |
| Were the reference standard results interpreted without knowledge of the results of the index test? | 0 | 0 | 0 | 0 | 0 | 0 |
| Were the same clinical data available when test results were interpreted as would be available when the test is used in practice? | 0 | 0 | 0 | 0 | 0 | 0 |
| Were uninterpretable/ intermediate test results reported? | 0 | 0 | 0 | 0 | 0 | 0 |
| Were withdrawals from the study explained? | 0 | 0 | 0 | 0 | 0 | 0 |
| Total | 10 | 10 | 10 | 9 | 9 | 10 |


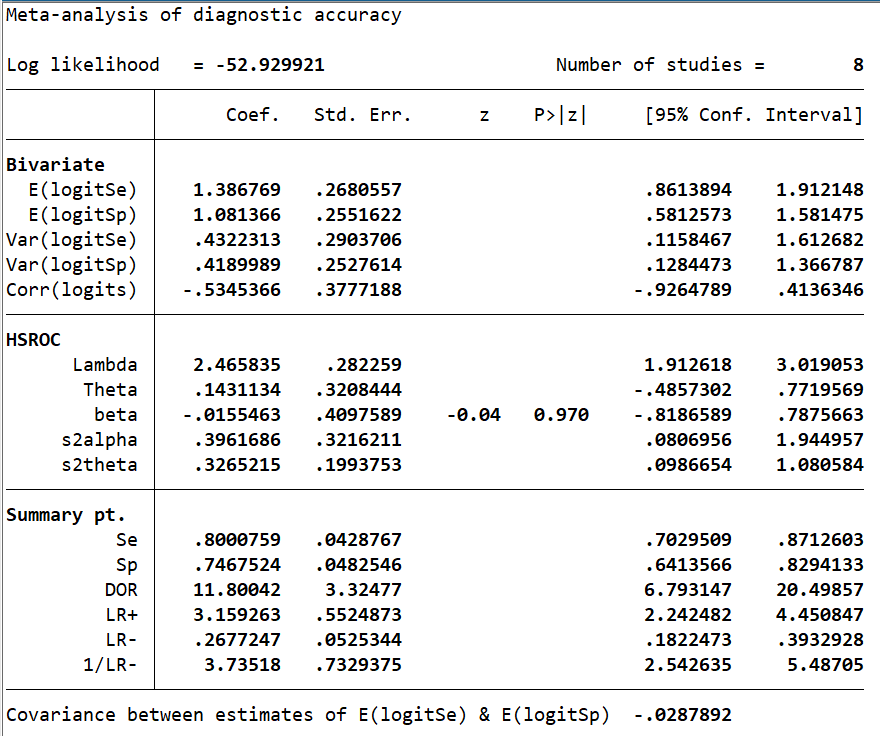


Supplementary Figure 2. Calculation of summary estimates.
